# Concurrent Central Diabetes Insipidus and Acute Myeloid Leukemia

**DOI:** 10.1155/2021/8898671

**Published:** 2021-02-16

**Authors:** Stephanie L. Pritzl, Daniel R. Matson, Mark B. Juckett, David J. Ciske

**Affiliations:** University of Wisconsin School of Medicine and Public Health, Madison, Wisconsin, USA

## Abstract

Central diabetes insipidus (CDI) is a rare reported complication of acute myeloid leukemia (AML). The onset of AML-associated CDI often precedes the diagnosis of AML by weeks or months and is considered an adverse prognostic indicator in this setting. The mechanism of AML-associated CDI is not known; however, it is often reported in the setting of cytogenetic events resulting in MDS1 and EVI1 complex locus protein (MECOM) gene overexpression. Here, we describe a case of new onset CDI which preceded a diagnosis of AML by 1 month. We detail the clinical and laboratory evaluation of the patient's CDI, and we describe the pathological and laboratory workup of their AML, which ultimately yielded a diagnosis of AML with myelodysplasia-related changes. Additional cytogenetic findings included the identification of the t (2;3)(p23;q27), which leads to MECOM gene overexpression and which to our knowledge has not previously been reported in the setting of AML-associated CDI. The patient received induction chemotherapy followed by allogeneic hematopoietic stem cell transplantation but experienced disease relapse and passed away nine months after initial diagnosis. We emphasize that new onset CDI can occur as a rare complication of AML where it portends a poor prognosis and may be related to AML subtypes displaying MECOM gene dysregulation.

## 1. Introduction

Central diabetes insipidus (CDI) as a complication of acute myeloid leukemia (AML) is rare, and the underlying mechanism(s) of AML-associated CDI remains incompletely understood. When it occurs, the onset of CDI typically precedes the diagnosis of AML by 1-2 months [1]. However, CDI may also occur at the time of AML diagnosis or as the initial manifestation of AML relapse [1, 2]. AML-associated CDI is hypothesized to represent an adverse prognostic indicator of AML, even when CDI symptoms are ameliorated by administration of desmopressin (DDAVP) [1–3]. Here, we present a case of CDI preceding an initial diagnosis of AML.

## 2. Case Presentation

A previously healthy 71-year-old man presented to his primary care physician with abrupt onset of polyuria and polydipsia. Physical exam was notable for intact visual fields and no evidence of hypovolemia. His medications included only aspirin (81 mg daily) and simvastatin (40 mg daily). Laboratory testing revealed leukopenia and macrocytic anemia (Table 1), in the setting of a previously normal complete blood count (CBC) one year prior. Comprehensive metabolic panel, hemoglobin A1c, and urinalysis were unremarkable. However, urine osmolality was inappropriately low in conjunction with an elevated serum osmolality (Table 1). Polyuria was confirmed with a 24-hour urine collection. An 8-hour water deprivation test was completed and demonstrated worsening hypernatremia with a suboptimal response in urinary concentration (Table 1). A subsequent desmopressin challenge was notable for decreased urine output and increased urine osmolality (Table 1). Levels of plasma vasopressin and copeptin, which comprises a portion of the vasopressin precursor molecule, were not measured. Other hormones dependent on pituitary function including serum prolactin, free T4, cortisol (AM), and free testosterone were all within normal limits. MRI head demonstrated lack of the normal posterior pituitary bright spot. The laboratory and imaging findings were consistent with CDI, and the, patient began treatment with DDAVP nasal spray which led to rapid improvement in his symptoms.

**Figure 1:**
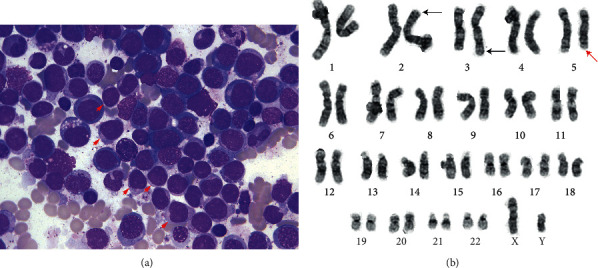
Bone marrow aspirate and AML cytogenetic karyotype: (a) wright-stained bone marrow aspirate showing increased blasts (examples highlighted by red arrowhead; 1000x original magnification); (b) AML karyotype demonstrating the t (2;3)(p23;q27) (black arrows) and del (5q)(q31;q33) (red arrow).

During this 21-day period, serial CBCs revealed a persistent leukopenia (WBC 3.4 K/*μ*L), worsening macrocytic anemia (Hb 11.7 g/Dl and MCV 107 fL), and a progressive thrombocytosis (PLT 500 K/*μ*L and normal 160–370 K/*μ*L). A manual differential performed on peripheral blood revealed 17% circulating blasts, and bone marrow biopsy demonstrated a hypercellular marrow virtually effaced by leukemic blasts (Figure 1(a)). Flow cytometry revealed a myeloid immunophenotype confirming a diagnosis of AML, without features worrisome for acute promyelocytic leukemia. Routine ancillary testing for newly diagnosed AML includes (minimally) the following studies performed on the leukemic blast population: karyotyping by classical cytogenetics, evaluation for core binding factor rearrangements by either fluorescence in situ hybridization (FISH) or classical cytogenetics, and mutational analysis of the fms-like tyrosine kinase 3 (FLT3) gene [4].

In this case, cytogenetic analyses revealed the t (2;3)(p23;q27), which is a chromosomal translocation often associated with MDS1 and EVI1 complex locus (MECOM) gene overexpression, as well as the del (5) q31;q33) (Figure 1(b)). FISH was negative for core binding factor rearrangements, and molecular analyses revealed no mutation in FLT3. Based on the presence of the del (5)(q31;q33), a final diagnosis of AML with myelodysplasia-related changes (AML-MRC) was rendered. AML-MRCs are a subtype of AML that are associated with a poor prognosis. They are diagnosed when AML patients have a known history of myelodysplastic syndrome, display significant morphologic dysplasia in the bone marrow at leukemic presentation, or when their AML harbors characteristic cytogenetic findings that are associated with myelodysplasia [5]. These characteristic cytogenetic findings include deletions in chromosome 5, as seen in our patient.

The patient received induction chemotherapy with liposomal daunorubicin and cytarabine which failed to reduce his bone marrow blast count. This was followed by two cycles of azacytidine and lenalidomide and reinduction with fludarabine, cytarabine, and granulocyte colony-stimulating factor. Three weeks following his second induction, the bone marrow blast count percentage was 95%. He then received an allogeneic stem cell transplantation (alloSCT) following pretreatment with an experimental radiolabeled anti-CD45 monoclonal antibody (IOMAB-01). Unfortunately, he experienced disease relapse 1 month after transplant and ultimately passed away 9 months after his initial diagnosis. Interestingly, prior to transplant, he required 40 mcg/day of DDAVP 0.01% nasal spray (20 mcg twice daily) to control his CDI symptoms, but following his transplant, he was able to space out the dosing of his DDAVP to every 4-5 days.

## 3. Discussion

AML-associated CDI is a rare but increasingly recognized complication of AML. In perhaps the largest study of AML-associated CDI to date, Ladigan et al. analyzed 51 reports of adults with myeloid malignancies and associated CDI [1]. They found a median age of diagnosis of 48 years, which is younger than the median age of 65 years reported across all new AML diagnoses [6]. At least 9 cases have also been reported in the pediatric population [7–13]. In addition, patients with AML-associated CDI show a female predominance (59% female), compared to a mild male predominance across all AMLs. The majority (45/51) of these cases occur in the setting of *de novo* AMLs, wherein the patient has no known prior diagnosis of a primary bone marrow neoplasm. The remaining cases are comprised of either myelodysplastic syndrome (MDS), which is a common precursor to AML, or AML transformed from aplastic anemia, MDS, or chronic myelomonocytic leukemia (CMML).

The diagnosis of AML-associated CDI is essentially identical to the diagnosis of CDI in any other setting [14, 15]. Most patients (75%) will be diagnosed with CDI concurrently with their AML diagnosis, with most remaining cases presenting no more than 2 months prior to or following their AML diagnosis [1]. While the majority of patients with AML-associated CDI (61%) will have no associated brain imaging abnormalities, some may have pathologic findings including loss of the posterior pituitary bright spot or nodular thickening/attenuation of the pituitary stalk [1]. MRI of our patient revealed absence of the posterior pituitary bright spot which can be indicative of hypothalamic pituitary dysfunction. The normal pituitary bright spot seen on T1-weighted MRI is thought to result from the T1-shortening effect of stored vasopressin in the posterior pituitary [16].

The pathogenesis of AML-associated CDI remains unclear although there are hints that dysmegakaryopoiesis and resulting platelet dysfunction could lie at the heart of this disorder. Previously, clinicians hypothesized that CDI occurred secondary to pituitary damage as a consequence of tumor-mediated destruction or infection. Early cohort studies of deceased patients with leukemia-associated CDI noted two types of lesions that could be routinely identified at autopsy in the supraopticohypophyseal system: (1) leukemic infiltrates and/or (2) thrombosis of small vessels [16]. However, it should be noted that leukemic infiltrates are not uncommonly seen at autopsy in AML patients who never developed CDI [2, 17, 18]. More recently, multiple case series have revealed a strong association between AML-associated CDI and cytogenetic abnormalities in the concurrent AML, chiefly inversions involving chromosome 3 (inv (3)) and monosomies involving chromosome 7 (monosomy 7) [1, 3, 19, 20]. Interestingly, at least a subset of cases with inv (3) harbor fusion events leading to MECOM gene overexpression, which has been shown to drive both leukemogenesis and dysmegakaryopoiesis [3, 21, 22]. It is theorized that the resulting abnormal platelets could adversely impact circulating vasopressin function, as 90% of peripheral vasopressin is platelet-bound [1–3]. Cytogenetic studies in our patient revealed the t (2;3)(p23;q27), a translocation that also typically results in MECOM overexpression and which to our knowledge has not been previously reported in the setting of AML-associated CDI [23]. Interestingly, our patient also showed a progressive thrombocytosis, an uncommon event in AML that has nonetheless been reported in the setting of the t (2;3) and MECOM overexpression [24]. Finally, our patient showed a greatly decreased requirement for DDAVP following alloSCT, after which his circulating platelets would have been derived from donor marrow. This decreased requirement for DDAVP and even complete resolution of CDI following AML treatment has been previously reported [1] and argues against pituitary destruction as the cause of AML-associated CDI in these cases.

Clinicians treating a patient with AML-associated CDI must correct the patient's CDI while also identifying an optimal treatment strategy for their AML. 90% of reported AML-associated CDI cases have a documented response to a vasopressin analog (mainly DDAVP), with the majority of patients demonstrating a marked improvement in CDI symptoms upon treatment [1]. Our patient's CDI symptoms are rapidly improved with DDAVP nasal spray. In comparison, treatment of the underlying AML depends on a number of factors, including the presence or absence of prognostically important cytogenetic and molecular findings and the patient's ability to tolerate therapy. In this respect, it is important to note that both monosomy 7 and inv (3) constitute unfavorable-risk cytogenetic findings in AML and are independently associated with worse rates of complete remission and overall survival [25–27]. Thus, independent of CDI, most AML-associated CDI patients would already be expected to have a 5-year AML-related survival of only 5-10%, compared to a 5-year survival of 29% across all AMLs [6]. In general, there is also a paucity of data regarding optimal induction regimens for AML patients with associated CDI. In 39 patients who received treatment with either standard-dose cytarabine and daunorubicin (often referred to as 7 + 3) or high-dose cytarabine, the rate of complete remission (CR) was only 15% [28–33]. Sufficient reports of other treatment regimens are not readily available. Perhaps not surprisingly given these data, AMLs in patients with AML-associated CDI confer a dismal prognosis, with an overall 1-year survival as low as 20% and a median overall survival time of only 6 months [1, 3]. However, CDI-associated AML patients who underwent alloSCT showed markedly better outcomes, with 40% surviving to 12 months. This is consistent with the survival benefit seen with alloSCT following first CR in AML patients with unfavorable risk cytogenetics [34]. Thus, the best available evidence suggests that patients with AML-associated CDI typically have unfavorable-risk cytogenetics and stand to benefit from intensive induction chemotherapy regimens followed (when possible) by an alloSCT in the first CR. Unfortunately, our patient never achieved a CR despite receiving multiple induction regimens, and although he underwent an alloSCT facilitated by pretreatment with an experimental therapy, he rapidly relapsed.

We reiterate that CDI is a rare but recognized complication of AML which is associated with a poor prognosis. We support future studies examining the consequences of MECOM gene dysregulation in AML and vasopressin function in both the pituitary and peripheral circulation.

## Figures and Tables

**Table 1:** Laboratory data.

Laboratory testing at diagnosis			
Assay	Result	Normal values	
WBC	2.3 K/*μ*L	3.8–10.5 K/*μ*L	
Hb	13.5 g/dL	13.6–17.2 g/dL	
MCV	101 fL	80–97 fL	
24-hour urine collection	12.5 L urine output	0.8–2.0 L	
Prolactin	8.9 ng/mL	3.5–19.4 ng/mL	
Cortisol (AM)	18.6 *μ*g/dL	10–20 *μ*g/dL	
Free T4	0.81 ng/L	0.70–1.48 ng/L	
Total testosterone	338 ng/dL	300–720 ng/dL	

8-hour water deprivation test			
Assay	0 hours	8 hours	Normal values

Serum sodium	144 mmol/L	149 mmol/L	136–145 mmol/L
Serum osmolality	302 mOsm/kg	308 mOsm/kg	278–298 mOsm/kg
Urine osmolality	123 mOsm/kg	235 mOsm/kg	50–1200 mOsm/kg

Desmopressin challenge			
Time	Urine output (mL)	Urine osmolality (mOsm/kg)	

12-1 PM	725	87	
1 PM	2 mcg DDAVP SubQ		
1-2 PM	160	341	
2-3 PM	80	467	

## Data Availability

No data sets were generated in the preparation of this report.
